# Risk factors for calciphylaxis in Chinese hemodialysis patients: a matched case-control study

**DOI:** 10.1080/0886022X.2021.1884094

**Published:** 2021-03-01

**Authors:** Yuqiu Liu, Xiaoliang Zhang, Xiaotong Xie, Xin Yang, Hong Liu, Rining Tang, Bicheng Liu

**Affiliations:** Institute of Nephrology, Zhong Da Hospital, Southeast University, School of Medicine, Nanjing, China

**Keywords:** Calciphylaxis, calcific uremic arteriolopathy, risk factors, hemodialysis, case-control study

## Abstract

**Introduction:**

Calciphylaxis is a rare but potentially fatal disease commonly occurred in dialysis patients. Despite some previous studies on risk factors for calciphylaxis, there is still a lack of data from Chinese population.

**Methods:**

The retrospective matched case–control study about calciphylaxis was performed in Zhongda Hospital affiliated to Southeast University. The case group involved 20 hemodialysis patients who were newly diagnosed with calciphylaxis from October 2017 to December 2018. The 40 noncalciphylaxis patients undergoing dialysis with the same age and duration of dialysis were randomly selected as controls.

**Results:**

Most of calciphylaxis patients were male and elderly, while overweight people were more susceptible to the disease. Although incidence of secondary hyperparathyroidism was higher in calciphylaxis patients, the differences in duration of elevated serum intact parathyroid hormone (iPTH) and its highest value did not reach statistical significance compared with controls. No significant difference in warfarin therapy was discernible between two groups. The univariate regression analysis indicated that male, score of use of activated vitamin D and its analogues, corrected serum calcium level, serum phosphate, Ca × P product, iPTH, albumin, and alkaline phosphatase (ALP) level were significantly associated with calciphylaxis. Elevated levels of serum phosphate (OR 4.584, *p* = 0.027) and ALP (OR 1.179, *p* = 0.036), decreased level of serum albumin (OR 1.330, *p* = 0.013) were independent risk factors after multivariate analysis.

**Conclusion:**

This is the first report of risk factors associated with calciphylaxis in China. Increased levels of serum phosphate and ALP, decreased level of serum albumin were vital high-risk factors for calciphylaxis in Chinese hemodialysis population.

## Introduction

Calciphylaxis, also known as calcific uremic arteriolopathy (CUA), is a serious life-threatening vascular disease characterized by systemic arteriolar medial calcification with intimal fibrosis and thrombosis [1,2]. These pathological changes cause tissue ischemic necrosis, which further leads to skin ulcers and necrosis of surrounding tissues, even gangrene in severe cases [1,3,4]. Affected areas are usually accompanied by unbearable pain. Calciphylaxis is previously considered to be a rare disease, but it has been reported and paid attention worldwide in the last decade. The disease mostly occurs in patients with end-stage kidney disease (ESKD) undergoing dialysis, and its prevalence reach up to 4% [5–7]. Skimpy disease awareness rate frequently leads to missed diagnosis, misdiagnosis, and delay in treatment with poor prognosis [8].

The etiology and pathogenesis of calciphylaxis remain unclear. Therefore, understanding high-risk factors for calciphylaxis can provide important clues for further exploration of the exact etiology, while it is critical to the development of prevention and treatment measures in future. Risk factors proposed in previous studies include ESKD, female, obesity, diabetes, autoimmune disorders, primary or secondary hyperparathyroidism, hypercalcemia, hyperphosphatemia, hypoproteinemia, elevated alkaline phosphatase (ALP), vitamin K deficiency, hypercoagulable state, warfarin therapy, application history of high doses of calcium or activated vitamin D and long-term use of glucocorticoids or immunosuppressant, subcutaneous injection of insulin or heparin, kidney transplant, iron overload and so on [3,6,9,10]. Due to the differences in race, region, medication habits and dialysis prescriptions, the results of risk factors studies in different countries are varied and controversial. In addition, although there are more than 700,000 dialysis patients, China's research on calciphylaxis is still in its infancy without risk factor analysis based on Chinese population [11]. Thus, current study used a retrospective matched case–control study to explore the characteristics and risk factors for calciphylaxis in Chinese hemodialysis patients.

## Materials and methods

### Research object

The medical records of maintenance hemodialysis patients who were newly diagnosed with calciphylaxis at Zhongda Hospital affiliated to Southeast University from October 1, 2017 to December 31, 2018 were retrospectively evaluated. The patients with clinically suspected calciphylaxis based on characteristic skin lesions such as painful purpura and ischemic ulcer required histopathological examination. Typical pathological manifestations were medial calcification and intimal fibroplasia of small arteries, extravascular calcium deposition or thrombosis of pannicular and dermal arterioles. A total of 20 patients were newly diagnosed with calciphylaxis (case group). Subsequently, contemporaneous patients receiving hemodialysis without calciphylaxis were randomly selected as control group. The controls were matched to cases in a 2:1 ratio by age (year, within ±3) and duration of hemodialysis (month, within ±20%) as matching factors. Patients in control group were alive at the time of the survey. The study protocol was approved by the Ethics Committee for Clinical Research of Zhongda Hospital Affiliated to Southeast University (Approval number: 2018ZDSYLL100-P01), and complied with the Declaration of Helsinki. All participants' informed consent was obtained.

### Data collection

Data regarding demographics, comorbidities, ESKD and dialysis-related characteristics, and medications were abstracted from the medical records. Considering that patients' treatments with activated vitamin D and its analogues are usually diverse, and it is hard to evaluate them uniformly. Hence, scoring analysis about them was performed according to the design in Table 1. Laboratory indicators were also collected, including hemoglobin, serum calcium, serum phosphate, serum intact parathyroid hormone (iPTH), 25-hydroxyvitamin D, serum alkaline phosphatase (ALP), serum albumin, triglycerides, total cholesterol, plasma glucose (fasting), glycated hemoglobin, ferritin, hypersensitive c-reactive protein (hs-CRP), and the like. Among them, the serum calcium level needed to be corrected based on the albumin content [12], and the formula was: corrected serum Ca concentration (mg/dL) = measured Ca concentration (mg/dL) + 0.8 × [4.0 － measured serum albumin concentration (g/dL)] . The time points for all data selection were as follows: in the case group, data at the time of diagnosis of calciphylaxis were selected; for controls without calciphylaxis, the last available data were used. Furthermore, the clinical process of each calciphylaxis patient was summarized. All data were collected by one investigator, and two additional investigators independently reviewed to confirm the accuracy of the data.

**Table 1.** Evaluation for the use of activated vitamin D and its analogues.

Score			Duration of medication			
			<1 year	1 ∼ 3 years	3 ∼ 5 years	≥5 years
			1 point	2 points	3 points	4 points
**Drug dosage**	Normal use	1 point	2	3	4	5
	Off-label use	2 points	3	4	5	6
	Combination of normal use and pulse therapy	3 points	4	5	6	7
	Combination of off-label use and pulse therapy	4 points	5	6	7	8

Normal use: Routine dosage specified in drug instructions. The recommended daily dose usually does not exceed 1.0 μg.

Off-label use: Drug dosage exceeds the specification in the instruction.

Pulse therapy: High-dose intermittent therapy that high-dose drugs are given for a short term.

### Statistical analysis

All data were analyzed using SPSS Statistics 23 (IBM, Armonk, NY, USA), while the charts were plotted with Prism 6 (GraphPad Software Inc.). Results were expressed as number (%) for categorical variables, as mean ± SD for normally distributed continuous variables and as median [interquartile range (IQR)] for continuous variables with skewed distribution. For differences between groups, discrete variables at baseline were compared by chi-square test, and continuous variables were compared by Student’s t-tests or Mann–Whitney U-test. Univariate logistic regression analysis was applied to calculate the odds ratio (OR) and 95% confidence interval (CI) to detect the association between risk factors and calciphylaxis. The covariates included in the multivariate model were selected from factors identified as significant by the univariate model. Two-sided *p*-values <0.05 were considered statistically significant.

## Results

### Clinical features of calciphylaxis patients

**Figure 1.**
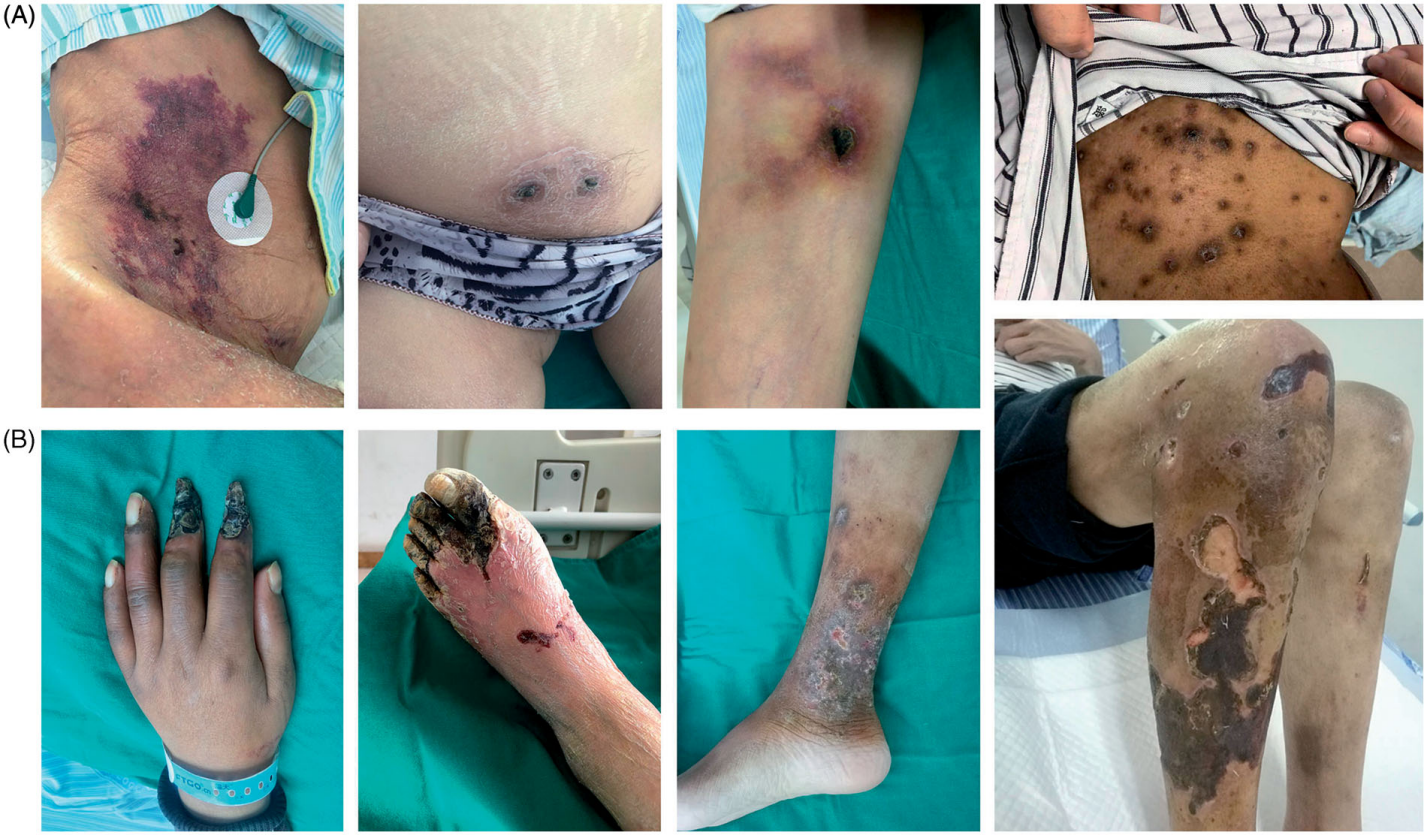
Clinical manifestations of calciphylaxis patients. (A) Central type, involve central areas in the subcutaneous adipose tissue, such as the abdomen or thighs. (B) Peripheral type, restrict to peripheral parts having limited adipose tissue, such as the digits and penis.

**Figure 2.**
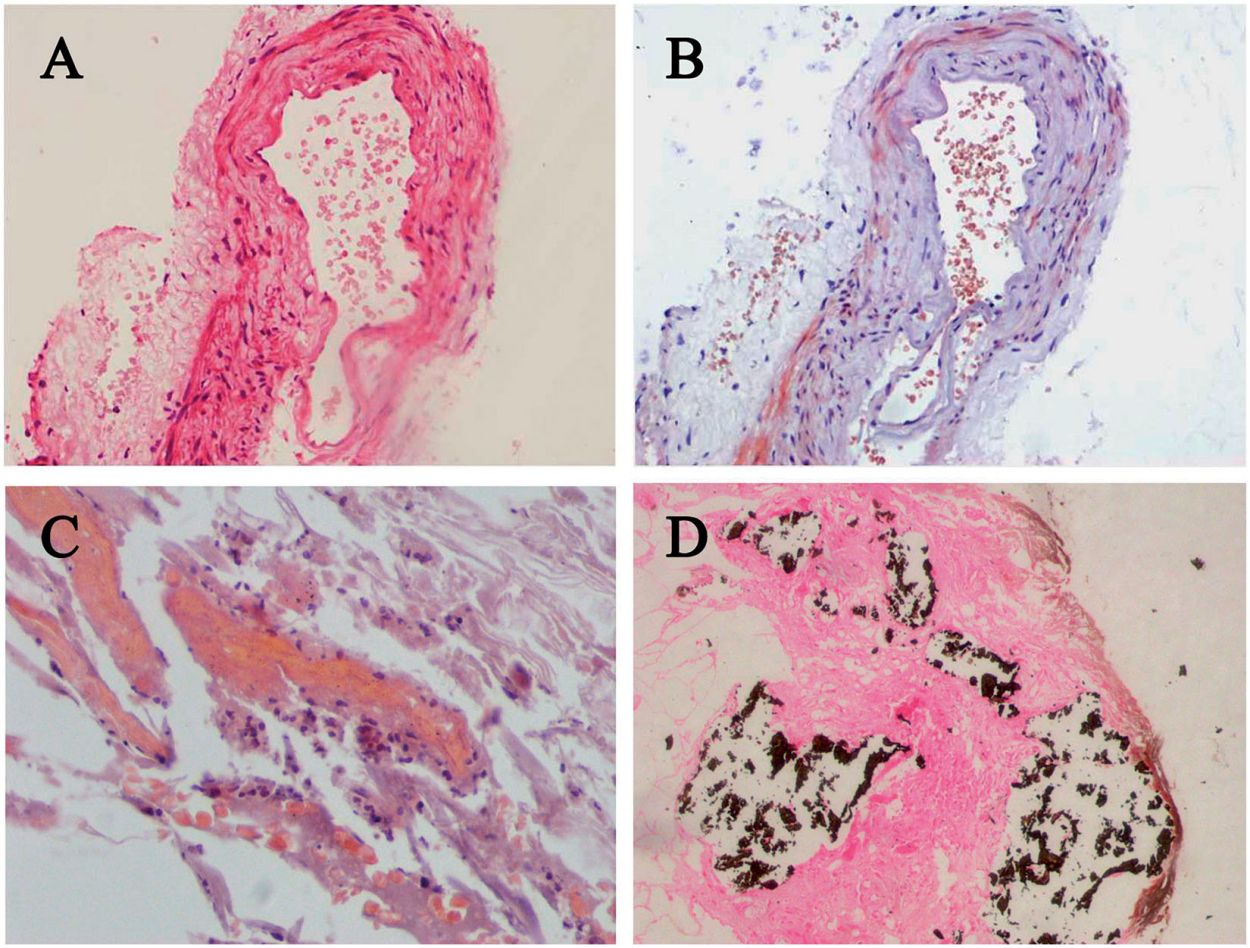
Histopathological characteristics of skin specimens in calciphylaxis patients. Calcification of the subcutaneous small vessel wall, accompanied by extensive calcium deposition of soft tissue in calciphylaxis patients. (A) H&E stain, (B–C) Alizarin Red Staining, (D) Von Kossa staining. Original magnification ×400.

20 hemodialysis patients were newly diagnosed with calciphylaxis identified by characteristic skin lesions (Figure 1) and histopathological features (Figure 2). Basic characteristics of calciphylaxis patients were tabulated in Table 2. Among them, 16 cases were male, accounting for 80%, while only four cases were female. The average age was 63.50 (42, 68.75) years old, and the elderly patients over 60-year-old reached 55% of the total. Body mass index (BMI) was lower than 18.5 kg/m^2^ in one patient (5%), within range from 18.5 to 24 kg/m^2^ in nine patients (45%) and higher than 24 kg/m^2^ in 10 patients (50%), suggesting that overweight patients were more likely to develop calciphylaxis. The average time interval since start of dialysis to calciphylaxis diagnosis was 114.65 ± 81.32 months. And the median time from the appearance of skin lesion to diagnosis was 6 (2, 15) months, as a result, most calciphylaxis patients had progressed to the terminal stage. According to the affected part of lesion, calciphylaxis can be classified as central type (involved central areas in the subcutaneous adipose tissue, such as the abdomen or thighs) or peripheral type (restricted to peripheral parts having limited adipose tissue, such as the digits and penis) [1,6]. The ratio of central type patients to peripheral type patients is 8 out of 12. As shown in Table 2, no statistic difference in gender, age distribution and BMI between two subgroups of calciphylaxis patients was observed. Twelve patients suffered from intolerable pain (NRS score ≥5), which was one of the features of this disease. In particular, pain was more common and pronounced in peripheral type calciphylaxis. All calciphylaxis patients received comprehensive treatment mainly with sodium thiosulfate, and 65% of the patients' condition deteriorated, in which 35% died, during the one-year follow-up period.

**Table 2.** Comparison of basic characteristics between central and peripheral calciphylaxis in hemodialysis patients.

Characteristic	Total (*N* = 20)	Central type (*N* = 8)	Peripheral type (*N* = 12)	*p*-value
Gender				
Male	16 (80%)	7	9	0.619
Female	4 (20%)	1	3	
Age (years)				
<20	0	0	0	0.493
20-40	4 (20%)	2	2	
40-60	5 (25%)	1	4	
≥60	11 (55%)	5	6	
BMI (kg/m^2^)				
<18.5	1 (5%)	0	1	0.428
18.5-24.0	9 (45%)	3	6	
≥24.0	10 (50%)	5	5	
Time interval since start of dialysis to calciphylaxis diagnosis (months)	114.65 ± 81.32	109.50 ± 77.09	118.08 ± 87.22	0.824
Time from the appearance of skin lesion to diagnosis (months)	6 (2, 15)	6 (4, 20)	5 (2, 11.5)	0.396
Pain (NRS score ^a^)	8 (5, 10)	3 (2, 6)	8.5 (6.25, 10)	0.039
Clinical prognosis (Follow-up for one year)				
Death	6 (30%)	3	3	0.867
Deterioration	7 (35%)	2	5	
Improvement	5 (25%)	2	3	
Healing	2 (10%)	1	1	

^a^ NRS score: Numerical rating scale, measures pain severity by asking the patient to select a number (from 0 to 10) to represent how severe the pain is.

BMI: Body mass index.

### Comparison of characteristics between calciphylaxis and noncalciphylaxis patients

A total of 60 hemodialysis patients were enrolled in present study: 20 patients in the case group and 40 as controls. As summarized in Table 3, the age [63.50 (42, 68.75) vs 63 (43, 70), *p* = 0.975] and the duration of dialysis (114.65 ± 81.32 vs 108.25 ± 74.26, *p* = 0.762) of two groups were well matched. But the proportion of female in the case group was lower than controls (20% vs 47.5%, *p* = 0.039). Calciphylaxis patients had the higher average BMI, but there was no significant difference between two groups. Besides, no significant difference was found between calciphylaxis patients and controls regarding the comorbidities of diabetes (45% vs 27.5%, *p* = 0.175), hypertension (90% vs 85%, *p* = 0.893), coronary heart disease (15% vs 15%, *p* = 1.000), atrial fibrillation (20% vs 10%, *p* = 0.502), liver disease (30% vs 22.5%, *p* = 0.527), chronic heart failure (35% vs 32.5%, *p* = 0.846), stroke (25% vs 20%, *p* = 0.912) or tumor (5% vs 10%, *p* = 0.656). Glomerulonephritis was the main cause of ESKD in both groups. Most patients were admitted to chronic kidney disease (CKD) stage 5 without any treatment, and only a few patients received immunosuppressive therapy (10% vs 7.5%, *p* = 1.000). No patients had received a kidney transplant.

**Table 3.** Comparison of baseline characteristics between hemodialysis patients who developed calciphylaxis and controls.

Characteristic	Cases (*N* = 20)	Controls (*N* = 40)	*p*-value
Gender			
Male	16 (80%)	21 (52.5%)	0.039
Female	4 (20%)	19 (47.5%)	
Age (years)	63.5 (42, 68.75)	63 (43, 70)	0.975
Duration of dialysis (months)	114.65 ± 81.32	108.25 ± 74.26	0.762
BMI (kg/m^2^)	23.55 ± 4.02	22.56 ± 3.79	0.354
Smoking	6 (30%)	11 (27.5%)	0.839
Comorbidities			
Diabetes	9 (45%)	11 (27.5%)	0.175
Hypertension	18 (90%)	34 (85%)	0.893
Coronary heart disease	3 (15%)	6 (15%)	1.000
Atrial fibrillation	4 (20%)	4 (10%)	0.502
Liver disease	6 (30%)	9 (22.5%)	0.527
Chronic heart failure	7 (35%)	13 (32.5%)	0.846
Stroke	5 (25%)	8 (20%)	0.912
Tumor	1 (5%)	4 (10%)	0.656
Secondary hyperparathyroidism			
iPTH ≥ 600 pg/mL	13 (65%)	19 (47.5%)	0.200
Duration of elevated iPTH (months)	32 (8.5, 66)	38 (15, 50)	0.715
iPTH’s highest value (pg/mL)	906.85 (327.90, 1496.35)	550.40 (169.50, 1098.23)	0.172
Cinacalcet treatment	10 (50%)	14 (35%)	0.264
Parathyroidectomy	5 (25%)	8 (20%)	0.912
Primary kidney disease			
Diabetic nephropathy	7 (35%)	6 (15%)	0.181
Glomerulonephritis	13 (65%)	32 (80%)	
Others	0	2 (5%)	——
Immunosuppressive therapy	2 (10%)	3 (7.5%)	1.000
Kidney transplant	0	0	——
Dialysate calcium concentration (1.25 mmol/L : 1.50 mmol/L : 1.75 mmol/L)	1:19:0	0:40:0	0.333
Kt/V	0.99 (0.82, 1.22)	0.97 (0.86, 1.13)	0.888
nPCR (g/kg/d)	0.85 ± 0.26	0.88 ± 0.25	0.704
Medication history			
Warfarin therapy	3 (15%)	2 (5%)	0.322
Insulin therapy	7 (35%)	6 (15%)	0.150
Drug score of activated vitamin D and its analogues	5 (3, 5.75)	3.5 (2, 5)	0.031
Calcium-containing phosphate binder	13 (65%)	21 (52.5%)	0.357
Noncalcium-containing phosphate binder	7 (35%)	15 (37.5%)	0.850
EPO	15 (75%)	33 (82.5%)	0.732
Iron agent	4 (20%)	12 (30%)	0.409
Laboratory examination			
Hemoglobin (g/L)	111.05 ± 26.95	108.25 ± 18.12	0.678
Serum calcium (mmol/L)	2.36 ± 0.27	2.28 ± 0.19	0.221
Corrected serum calcium (mmol/L)	2.46 ± 0.28	2.30 ± 0.20	0.015
Serum phosphate (mmol/L)	2.22 ± 0.52	1.69 ± 0.58	0.001
Ca × P product (mg/dL)	65.10 ± 17.70	47.93 ± 17.73	0.001
iPTH (pg/mL)	675.85 (298.20, 1496.35)	236.75 (85.03, 530.48)	0.003
Serum albumin (g/L)	35.15 ± 5.36	38.93 ± 4.50	0.006
ALP (IU/L)	149.5 (117.5, 289.25)	87 (63, 141)	0.001
Triglycerides (mmol/L)	1.29 (1.01, 2.68)	1.18 (0.75, 1.74)	0.111
Total cholesterol (mmol/L)	4.16 ± 1.37	3.68 ± 0.87	0.170
Plasma glucose (fasting) (mmol/L)	5.41 (4.66, 7.69)	4.86 (4.46, 5.49)	0.168
Ferritin (ug/L)	127.80 (35.05, 687.80)	111.05 (51.38, 303.43)	0.901
hs-CRP (mg/L)	14.3 (5.72, 37.05)	6.11 (3.58, 14.78)	0.037
ESR (mm/h)	27.50 (14.25, 69.75)	29.00 (19.75, 50.25)	0.958
INR	1.44 ± 1.46	1.07 ± 0.08	0.277

BMI: Body mass index; iPTH: Serum intact parathyroid hormone; Kt/V: Urea clearance index; nPCR: Normalized protein catabolic rate; EPO: Erythropoietin; ALP: Serum alkaline phosphatase; hs-CRP: Hypersensitive c-reactive protein; ESR: Erythrocyte sedimentation rate; INR: International normalized ratio.

**Figure 3.**
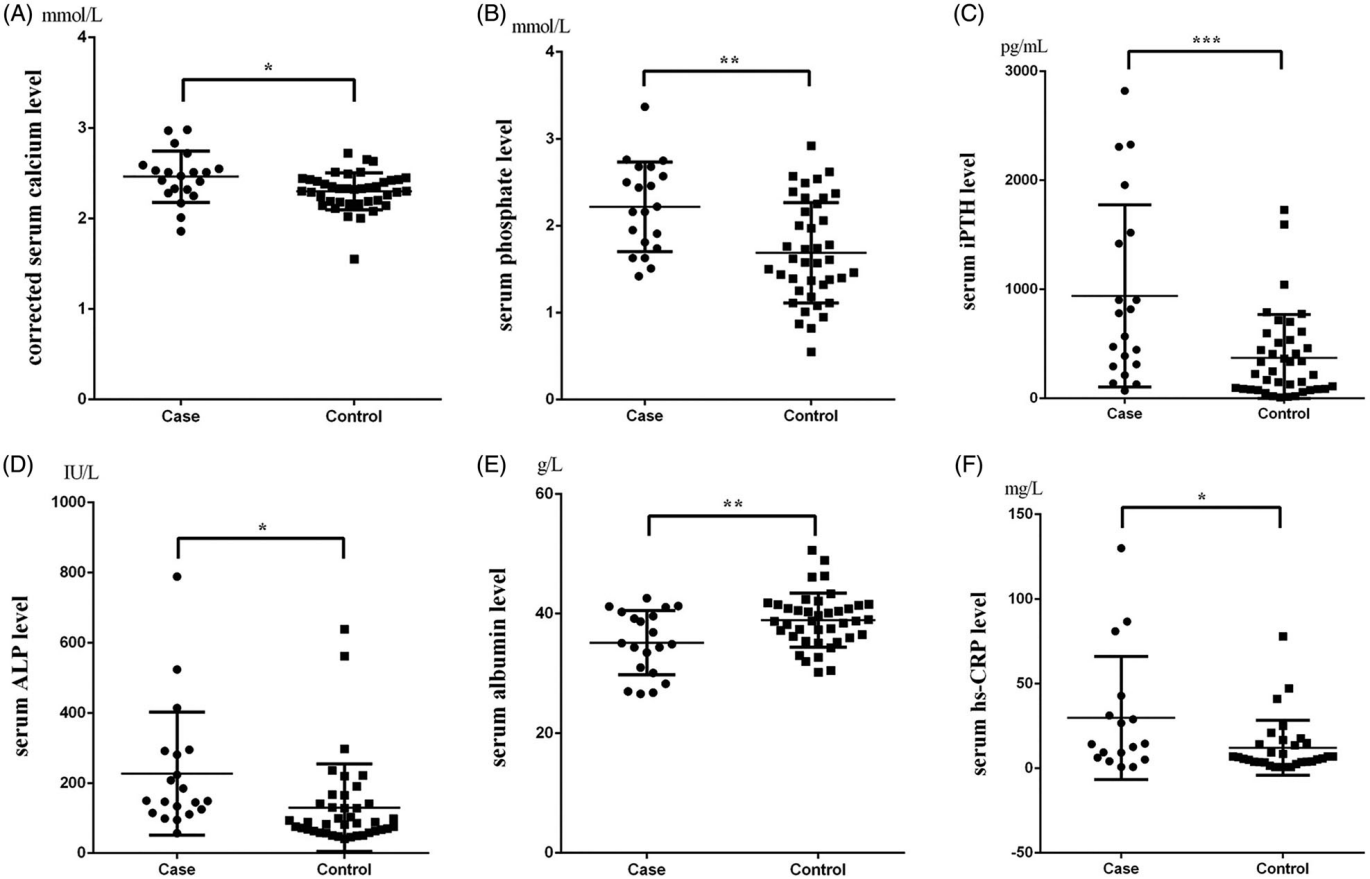
Scatter plots of laboratory examination from calciphylaxis patients and controls. Scatter plots of corrected serum calcium level (A), serum phosphate level (B), serum intact parathyroid hormone (iPTH) level (C), serum alkaline phosphatase (ALP) level (D), serum albumin level (E) and serum hypersensitive c-reactive protein (hs-CRP) level (F) of two groups. Case group selected the data at the time of diagnosis of calciphylaxis, and control group used the last available data. *p < 0.05, **p < 0.01 and ***p < 0.001 in comparison with the controls.

Secondary hyperparathyroidism (SHPT) has been extensively recognized as an imperative feature of chronic kidney disease-mineral and bone disorder (CKD–MBD), which can cause ectopic calcification of vascular and tissue in dialysis patients. In the current study, although the incidence of SHPT (iPTH ≥600 pg/mL) in calciphylaxis patients was as high as 65%, the differences in the incidence of SHPT, the duration of elevated iPTH and its highest value did not reach statistical significance compared with control group. It should be noticed that the case group used activated vitamin D and its analogues [5 (3, 5.75) vs 3.5 (2, 5), *p* = 0.031] at a higher dose or for a longer period. Warfarin therapy that is widely used in western countries is considered as a vital high-risk factor for calciphylaxis [13,14]. Since the exposure rate of warfarin in Chinese population was low, the difference did not reach statistical significance (15% vs 5%, *p* = 0.322). No significant difference in both calcium-containing (65% vs 52.5%, *p* = 0.357) and noncalcium-containing (35% vs 37.5%, *p* = 0.850) phosphate binder treatments was observed between two groups. What is more, the laboratory examination results showed that there were significant differences in corrected serum calcium (2.46 ± 0.28 vs 2.30 ± 0.20, *p* = 0.015), serum phosphate (2.22 ± 0.52 vs 1.69 ± 0.58, *p* = 0.001), Ca × P product (65.10 ± 17.70 vs 47.93 ± 17.73, *p* = 0.001), iPTH [675.85 (298.20, 1496.35) vs 236.75 (85.03, 530.48), *p* = 0.003], serum albumin (35.15 ± 5.36 vs 38.93 ± 4.50, *p* = 0.006), serum ALP [149.5 (117.5, 289.25) vs 87 (63, 141), *p* = 0.001] and hs-CRP [14.3 (5.72, 37.05) vs 6.11 (3.58, 14.78), *p* = 0.037] between case group and controls. However, scatter plots (shown in Figure 3) revealed several laboratory examination results from calciphylaxis patients were highly discrete with extreme values, especially serum iPTH and hs-CRP levels.

**Figure 4.**
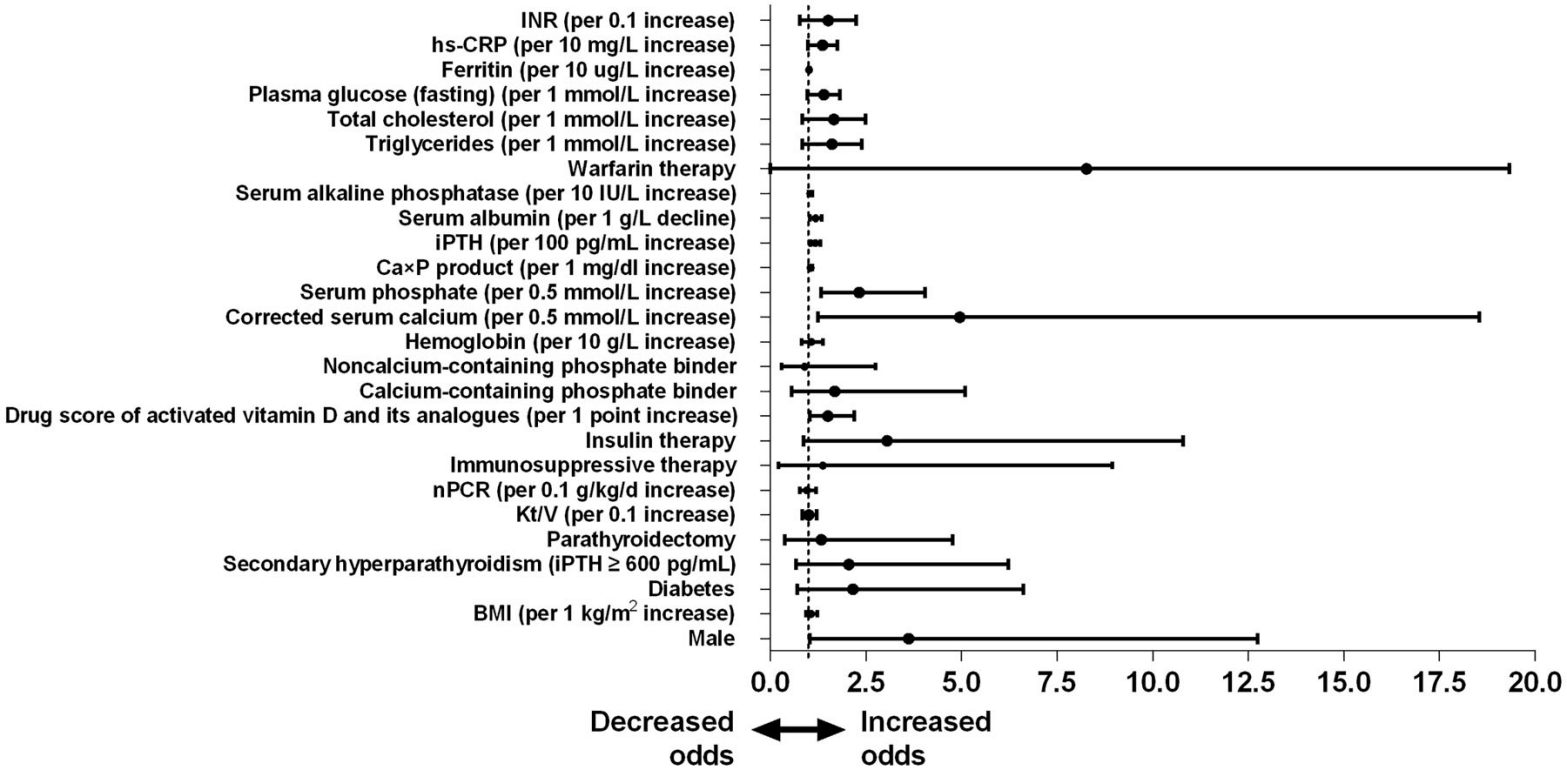
Forest plot of odds ratios of risk factors for calciphylaxis development based on univariate logistic regression analyses. Univariate logistic regression model showed odds ratios (ORs) of calciphylaxis development by patient characteristics at the time of diagnosis. Filled rounded denotes point estimate of ORs and error bars represent 95% confidence interval (CI). BMI: Body mass index; iPTH: Serum intact parathyroid hormone; Kt/V: Urea clearance index; nPCR: Normalized protein catabolic rate; hs-CRP: Hypersensitive c-reactive protein; INR: International normalized ratio.

### Risk factors for calciphylaxis in hemodialysis patients

The risk factors related with calciphylaxis development screened by univariate logistic regression analysis were shown in Table 4 and Figure 4. Gender was of particular concern, and male was significantly associated with calciphylaxis (OR 3.619, 95% CI 1.027–12.748, *p* = 0.045). Upon univariate analysis, each one point increase in the score of use of activated vitamin D and its analogues (OR 1.505, 95% CI 1.029–2.201, *p* = 0.035) was associated with increased odds of calciphylaxis. But no correlation was found between calciphylaxis and other medication, such as warfarin, insulin, phosphate binder and iron agent. In terms of laboratory examination, each 0.5 mmol/L increase in corrected serum calcium level (OR 4.948, 95% CI 1.253–19.542, *p* = 0.023), each 0.5 mmol/L increase in serum phosphate level (OR 2.320, 95% CI 1.329–4.048, *p* = 0.003), each 1 mg/dL increase in Ca × P product (OR 1.054, 95% CI 1.018–1.091, *p* = 0.003), each 100 pg/mL increase in iPTH level (OR 1.173, 95% CI 1.048–1.312, *p* = 0.005), each 1 g/L decline in serum albumin level (OR 1.181, 95% CI 1.040–1.341, *p* = 0.010), each 10 IU/L increase in serum ALP level (OR 1.046, 95% CI 1.003–1.092, *p* = 0.036) and each 10 mg/L increase in hs-CRP level (OR 1.331, 95% CI 1.000–1.772, *p* = 0.050) at the time of diagnosis were all associated with calciphylaxis. In addition, there were no significant relevance between calciphylaxis and other covariates.

**Table 4.** Results from univariate and multivariate logistic regression analyses to identify risk factors of calciphylaxis in hemodialysis patients.

Characteristic	Univariate analysis		Multivariate analysis	
	*p*-value	OR (95% CI)	*p*-value	OR (95% CI)
Male	0.045	3.619 (1.027–12.748)	0.096	10.328 (0.662–161.115)
BMI (per 1 kg/m^2^ increase)	0.349	1.069 (0.930–1.230)		
Diabetes	0.179	2.157 (0.703–6.621)		
Secondary hyperparathyroidism (iPTH ≥ 600 pg/mL)	0.204	2.053 (0.677–6.221)		
Parathyroidectomy	0.658	1.333 (0.373–4.770)		
Immunosuppressive therapy	0.742	1.370 (0.210–8.943)		
Kt/V (per 0.1 increase)	0.961	1.005 (0.838–1.205)		
nPCR (per 0.1 g/kg/d increase)	0.699	0.958 (0.771–1.191)		
Medication history				
Warfarin therapy	0.207	3.353 (0.512–21.938)		
Insulin therapy	0.084	3.051 (0.862–10.799)		
Drug score of activated vitamin D and its analogues (per 1 point increase)	0.035	1.505 (1.029–2.201)	0.109	2.042 (0.852–4.893)
Calcium-containing phosphate binder	0.359	1.680 (0.554–5.092)		
Noncalcium-containing phosphate binder	0.850	0.897 (0.293–2.750)		
Laboratory examination				
Hemoglobin (per 10 g/L increase)	0.629	1.066 (0.823–1.379)		
Corrected serum calcium (per 0.5 mmol/L increase) ^*^	0.023	4.948 (1.253–19.542)		
Serum phosphate (per 0.5 mmol/L increase)	0.003	2.320 (1.329–4.048)	0.027	4.584 (1.185–17.726)
Ca × P product (per 1 mg/dL increase)	0.003	1.054 (1.018–1.091)		
iPTH (per 100 pg/mL increase)	0.005	1.173 (1.048–1.312)	0.123	0.817 (0.632–1.056)
Serum albumin (per 1 g/L decline)	0.010	1.181 (1.040–1.341)	0.013	1.330 (1.063–1.664)
ALP (per 10 IU/L increase)	0.036	1.046 (1.003–1.092)	0.036	1.179 (1.011–1.376)
Triglycerides (per 1 mmol/L increase)	0.121	1.485 (0.900–2.448)		
Total cholesterol (per 1 mmol/L increase)	0.114	1.517 (0.905–2.542)		
Plasma glucose (fasting) (per 1 mmol/L increase)	0.061	1.349 (0.987–1.845)		
Ferritin (per 10 ug/L increase)	0.167	1.011 (0.996–1.026)		
hs-CRP (per 10 mg/L increase)	0.050	1.331 (1.000–1.772)		
INR (per 0.1 increase)	0.196	1.390 (0.843–2.292)		

*Since serum albumin concentration was used in the calculation of corrected serum calcium concentration, they could not be simultaneously included in the multivariate logistic analysis. When corrected serum calcium concentration was included instead of albumin concentration, corrected serum calcium concentration was found not to be a significant risk factor either. And Ca × P products also didn’t enter the multivariate analysis.

BMI: Body mass index; iPTH: Serum intact parathyroid hormone; Kt/V: Urea clearance index; nPCR: Normalized protein catabolic rate; ALP: Serum alkaline phosphatase; hs-CRP: Hypersensitive c-reactive protein; INR: International normalized ratio.

The covariates identified by the univariate model were included in the multivariate model, and Table 4 also showed the results of multivariate logistic regression analysis. Serum phosphate, serum albumin and serum ALP were still significantly associated with calciphylaxis, which were its independent risk factors. Each 0.5 mmol/L increase in serum phosphate level at the time of diagnosis increased the risk of calciphylaxis by 4.584-fold. And each 10 IU/L increase in serum ALP level, the risk was increased by 1.179-fold. Likewise, there was a 1.330-fold higher risk of the disease for each 1 g/L decline in serum albumin level. Although male, drug score of activated vitamin D and its analogues, iPTH level also entered the multivariate formula, they were not significant risk factors.

## Discussion

Although there have been some sporadic case reports of calciphylaxis in China, this is the first study about the risk factors associated with calciphylaxis based on Chinese hemodialysis population. The final results of the matched case-control study suggested that elevated levels of serum phosphate and serum alkaline phosphatase, decreased level of serum albumin were important high-risk factors for calciphylaxis. An in-depth understanding of the risk factors is helpful to improve the level of clinical diagnosis and treatment for calciphylaxis.

In a previous nationally representative study across the United States, calciphylaxis was found to have a predilection for women [6], but on the contrary, we observed that most diseases occurred in Chinese male. In addition to certain selection bias, the high proportion of men among Chinese hemodialysis population may also cause an imbalance in the proportion of male and female patients. Moreover, racial factor is probably another imperative reasons for this result. The majority of patients in current study were diagnosed with calciphylaxis for at least six months after the skin lesions appeared, and they were in advanced stage of the disease. The delayed diagnosis caused by clinicians’ insufficient understanding of calciphylaxis contributes to this serious illness condition. The predilection sites of calciphylaxis are soft and fatty tissues, for instance, the abdomen, buttocks and thighs [1,4], although necrosis of the extremities such as fingers [15] and penises [16,17] is also constantly reported. Davis *et al.* conducted a literature review and found that obesity was identified as a risk factor in six of the eight studies reviewed [18]. Obesity was associated with central CUA, and it might increase the risk of calciphylaxis by 4-fold [19]. Even though BMI was ultimately not a statistically significant factor in present study, the average BMI level of calciphylaxis patients was notably higher than that in control group, which was in agreement with the previous findings. One reason for aforementioned result is that the degree of obesity in China's CKD population is far lower compared with western populations, and almost no calciphylaxis patient with BMI of more than 30 kg/m^2^ was included in this research [8,20]. On the other hand, BMIs of central calciphylaxis patients were extremely high or low, and malnutrition caused by the disease might offset part of the obesity status.

SHPT has long been considered as a momentous inducement of ectopic calcification in uremia patients. We noticed that SHPT was more prevalent among calciphylaxis patients compared with those without calciphylaxis development. In a randomized controlled trial involving more than 3800 hemodialysis patients, the incidence of calciphylaxis was prominently reduced in the cinacalcet-administered group [21,22]. Present study also confirmed that the median level of iPTH in the calciphylaxis group was 675.85 pg/mL, which was significantly higher than 236.75 pg/mL in controls. Nevertheless, the optimal iPTH level in calciphylaxis patients is uncertain, but extreme values (extremely high or low) should be avoided [1]. Parathyroidectomy (PTX) in calciphylaxis patients remains controversial, that the effect of it on survival is inconsistent in different studies [23]. Karmegam *et al.* reported a new case of calciphylaxis after PTX, that might be related to the rapid decline of iPTH [24]. After PTX, the original high transport state of the bone is affected by the decline of iPTH, and even low turnover bone disease may occur. Excessive calcium and phosphate would deposit in the vascular wall, making the medial membrane and soft tissues prone to calcification that may aggravate or induce calciphylaxis [24,25]. Therefore, cinacalcet, which could improve bone markers and stabilize vascular calcification, may be preferable to PTX for calciphylaxis patients with SHPT, and PTX is reserved for refractory cases [26–29].

When renal insufficiency occurs, reduced concentrations of activated vitamin D further contribute to SHPT [30]. Hence, activated vitamin D and its analogs which are commonly used in the treatment for SHPT in CKD patients can effectively reduce iPTH levels, improve the bone damage of osteoporosis and high transport bone disease in SHPT patients. Whereas, a series of side effects such as hypercalcemia and vascular calcification may occur during the treatment [30–32]. We found that the incidence of calciphylaxis was higher in patients with long-term or high-dose use of activated vitamin D and its analogues. In consequence, the uses of these drugs need to start with a low dose, then be adjusted according to the response of iPTH, and a risk/benefit ratio assessment is necessary. Furthermore, the levels of serum calcium, serum phosphate and serum ALP in calciphylaxis group were significantly increased. The potential risk factors observed in current study may be instructive to understand the pathobiology and pathogenesis of calciphylaxis. Multivariate logistic analysis showed that corrected serum calcium level was not an important risk factor, while serum phosphate and serum ALP levels were proved to be independent high-risk factors, which was another feature of our study. Previous studies had shown that extracellular phosphate (Pi) directly regulated the ability of vascular smooth muscle cells (VSMCs) to initiate matrix mineralization [33]. Pi alone was sufficient to cause senescence of VSMCs and further accelerated the formation of calcification [34]. Thus, the balance of Pi may be a key factor in regulating various vascular calcifications, including calciphylaxis [35].

In the initial stages of calcification, microcalcification is accompanied by persistent inflammation [36]. It is also thought that inflammation is a culprit for vascular calcification in atherosclerosis and diabetes [37]. This study noticed that hs-CRP levels in calciphylaxis patients were twice as high as that in those without calciphylaxis, suggesting there might be persistent microinflammation. Our another study reported that extracellular vesicles produced by macrophages aggregated in diameters and served as nucleating foci for mineralization in the section of skin tissue from calciphylaxis patients, which highlighted the contribution of inflammation to microcalcification [38]. As an important risk factor for calciphylaxis, a significant decrease in albumin levels had been observed in calciphylaxis patients with typical malnutrition in multiple studies [39–41]. What is more, protein-energy malnutrition and inflammation are usually concurrent in maintenance dialysis patients, so the concept of malnutrition-inflammation complex syndrome (MICS) is proposed [42]. MICS may provide a new insight into calcific disease in CKD [43,44].

Warfarin-associated calciphylaxis is a crucial clinical subgroup with independent pathophysiologic features [45]. Warfarin can not only promote vascular calcification by inhibiting the vitamin K-dependent matrix Gla protein which prevents calcium deposition in arteries, but also directly increase thrombosis through the coagulation pathway [45,46]. And the latest research observed that the ESKD undergoing hemodialysis is a deficiency state of vitamin K [47]. The vital effect of warfarin in calciphylaxis development was not observed in this study, which might be related to the low exposure rate of warfarin in China. Warfarin has been reported to be used in only 13.9% of Chinese non-valvular atrial fibrillation patients, and compliance is low [48]. In the future, prospective control studies can be used to further determine the role of warfarin therapy or oral vitamin K in calciphylaxis.

In summary, present study provided insights into the risk factors of calciphylaxis in Chinese hemodialysis population for the first time, which revealed high levels of serum phosphate and ALP, low level of serum albumin at the time of diagnosis are its independent risk factors. Unlike previous studies in western countries, female and warfarin therapy did not show an increased risk for calciphylaxis development in our research. Therefore, the results of risk factor analysis are bound up with various factors, such as race, prescribing habits, etc. It is necessary to explore local risk factors of calciphylaxis based on indigenous conditions, which is more realistic and more urgent. Nevertheless, considering the statistical limitations of this study due to the small sample size, the evidence is inadequate. In future, large-scale multicenter epidemiological studies are needed to clarify high-risk factors and explore the pathogenesis of calciphylaxis in China.
